# Meta-Analysis on the Association of Neuropeptide Y rs16139 Variant With the Risk of Alcoholism

**DOI:** 10.3389/fpsyt.2021.737440

**Published:** 2021-10-28

**Authors:** Biqing Chen, Manish Yadav, Madhubala Mulkalwar, Lakkakula Saikrishna, Henu Verma, Weibing Ye, L. V. K. S. Bhaskar

**Affiliations:** ^1^Department of Sports Operation and Management, Jinhua Polytechnic, Jinhua, China; ^2^Department of Zoology, Guru Ghasidas Vishwavidyalaya, Bilaspur, India; ^3^Department of Pathology, Shri Shankaracharya Institute of Medical Sciences (SSIMS), Bhilai, India; ^4^Department of Public Health, Nellore Municipal Corporation, Nellore, India; ^5^Department of Immunopathology, Institute of Lungs Biology and Disease, Comprehensive Pneumology Center, Helmholtz Zentrum, Munich, Germany; ^6^Exercise and Metabolism Research Center, College of Physical Education and Health Sciences, Zhejiang Normal University, Jinhua, China

**Keywords:** NPY, rs16139, alcoholism, meta-analysis, association

## Abstract

**Introduction:** The neuropeptide-Y (NPY) is involved in the development of alcoholism through NPY receptors. A T>C mutation causes substitution of leucine to proline at codon 7 (L7P; rs16139) in the signal peptide of neuropeptide Y is known to cause a 42% increase in plasma NPY levels. Studies that analyzed the association between *NPY* rs16139 and alcoholism risk did not demonstrate conclusive evidence for this relationship. The present study aims to evaluate the association between *NPY* gene rs16139 variant and alcohol dependence.

**Method:** An electronic search of databases including PubMed and Google Scholar was performed to retrieve studies investigating the association between *NPY* rs16139 and alcoholism. The pooled odds ratio (OR) with 95% confidence interval (CI) was calculated in allelic and dominant genetic models. Sensitivity analyses and publication bias were assessed in our meta-analysis. The meta-analysis was conducted using the MetaGenyo web tool.

**Result:** Significant heterogeneity was observed across studies (*p* < 0.001). Our results have shown that there is no significant association between *NPY* rs16139 variant and the risk of alcoholism in allelic (OR = 0.98, 95% CI 0.70–1.38, *p* = 0.921) and dominant models (OR = 0.98, 95% CI 0.69–1.40, *p* = 0.919). Begg's funnel plot and Egger's test have not shown publication bias (*p* = 0.332).

**Conclusion:** To the best of our knowledge, this is the first meta-analysis that evaluates the relationship between the *NPY* rs16139 polymorphism and the risk of alcoholism. Our large-scale meta-analysis suggests that *NPY* rs16139 polymorphism is not associated with alcoholism. However, further studies are needed to increase our understanding of the relationship between *NPY* variants in alcoholism.

## Introduction

Alcohol is one of the most extensively used psychoactive drugs, which has become a part of the culture in many societies. In recent years, alcohol consumption has been rapidly increased worldwide and is responsible for social and medical problems (1). Alcohol use disorder is a chronic, recurrent disease with significant social implications. The family and twin studies suggested a 40 and 60% risk rate related to heredity (2). Genetic predisposition, environmental factors, stress, mental health, age, and gender of the patient are important risk factors for alcoholism (3). Especially, genetic risk factors play a key role in the etiology of alcoholism. Thus, it is essential to understand the genetic basis of alcoholism in order to ascertain an individual's risk of alcohol use disorder and develop effective treatment and prevention programs. Molecular genetic studies to identify the association of genes with alcoholism suggested that many candidate genes such as *ADH1B, ALDH2, CHRM2, DRD2, GABRA2, OPRM1, NPY*, and *SLC10A2* are associated with alcoholism (4–7).

Numerous lines of evidence suggested that the Neuropeptide Y is an angiogenic neurotransmitter whose physiological and behavioral effects are mediated by its receptor subtypes (Y1–Y5) (8, 9). The NPY is highly expressed in the hypothalamus, specifically in the arcuate and paraventricular nuclei, and is involved in energy homeostasis, memory function, and plasticity (10). Neuropeptide Y (NPY) is an evolutionarily conserved neuropeptide that participates in many physiological functions (11). The link between NPY and alcohol consumption has been studied primarily on animals (12, 13). It is well-known that excessive alcohol consumption is frequently associated with anxiety and depression. The amygdala, a region located deep inside the temporal lobe is known express NPY and is associated with positive and negative emotional effects in healthy subjects (14, 15). The effects of NPY on alcohol-related behaviors have been attributed to their modulation of excitatory and inhibitory transmission in the amygdala and neighboring regions (11). Alcohol consumption was reduced in mice overexpressing NPY, but increased in mice deficient in NPY (12). In addition, a reduction in ethanol consumption was observed when alcohol-preferring rats were injected with NPY (16). Hence, manipulating the NPY system appears to be a promising target for combating the neural alterations, alcohol use disorder related behaviors, and cognitive deficits caused by many drugs (17).

The gene coding for human NPY is located at the 7p15.1 locus of the 7th chromosome (18). A change in T>C nucleotide at 1,128 is leading to a change in amino acid leucine to proline at codon 7 (L7P) in the signal peptide of neuropeptide Y (19). The substitution of proline for leucine results in a 42% increase in plasma NPY levels (20). The association between *NPY* gene variant and alcoholism has been demonstrated in large cohorts of alcohol users and veterans (21). A promoter SNP, rs16147, alters expression of NPY *in vitro* and seems to contribute for >50% of the variation in expression *in vivo* (22). Further, the link between the NPY rs16139 polymorphism and the risk of alcoholism has been studied in many populations. However, the results are not conclusive (23–26). To date, no meta-analysis has been conducted to investigate the relationship between the NPY gene rs16139 polymorphism and the risk of alcohol use disorders. However, in the present study, we conducted a meta-analysis to evaluate the strength of association between NPY rs16139 variant and the risk of alcoholism.

## Materials and Methods

### Search Strategy and Selection Criteria

**Figure 1:**
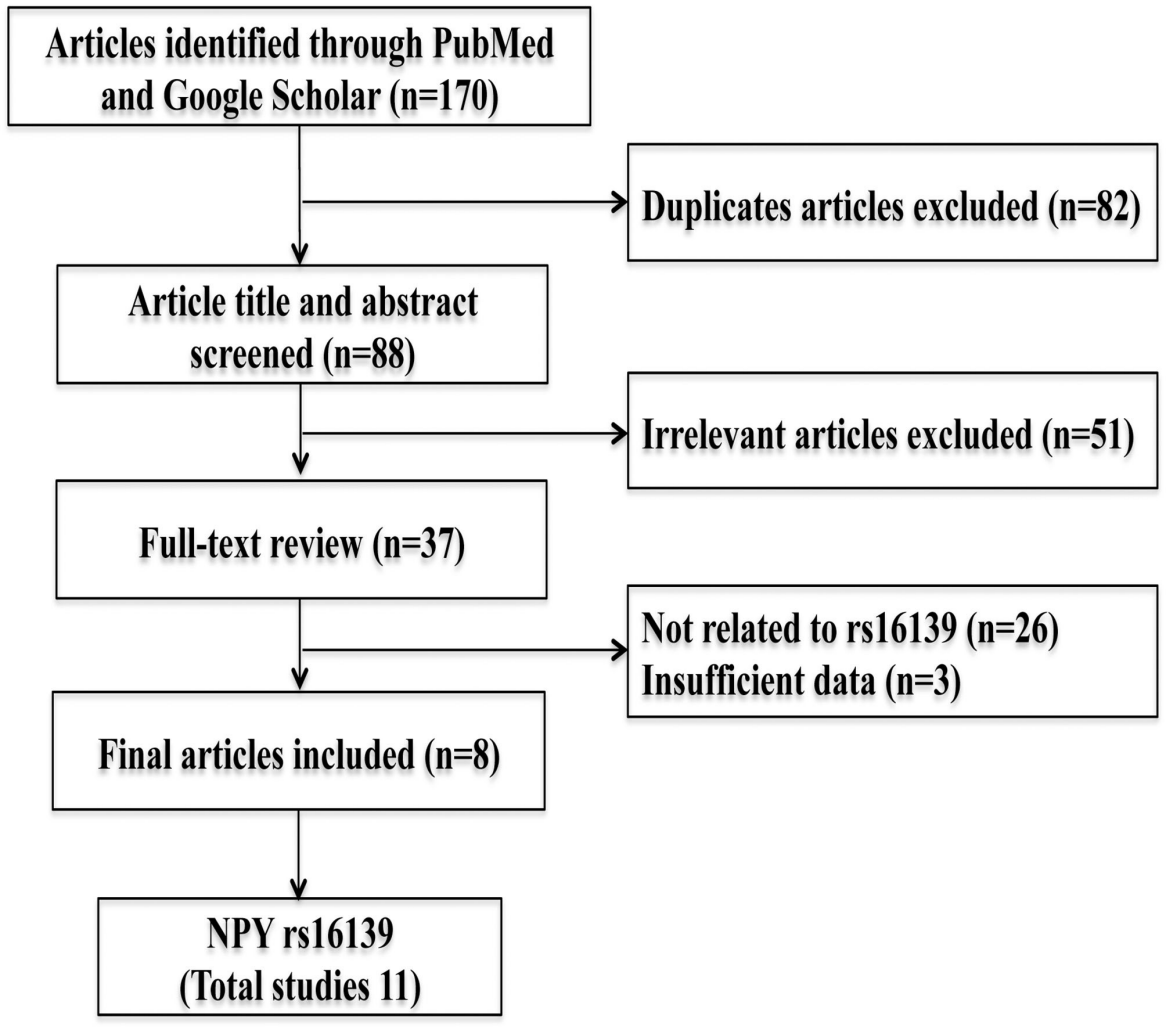
Flowchart depicting the article selection criteria.

According to the PRISMA guidelines (Figure 1), all studies examining the association of NPY gene rs16139 with alcoholism have been identified and summarized (27). PubMed, Web of Science and Google Scholar databases were searched using a combination of keywords like Neuropeptide Y (NPY), Leu7Pro, T1128C, rs16139, alcoholism, and alcohol dependence. The studies were eligible for inclusion if they met the following criteria: (1) case-control studies assessing *NPY* rs16139 polymorphisms and alcoholism risk (2) Studies having rs16139 genotypes for estimating the odds ratio. Studies with no specific control group and no detailed genotyping data for calculating odds ratios and 95% confidence intervals have been excluded. From each paper, first author, year of publication, country, genotypes from both alcoholism and control groups were extracted and tabulated (Table 1). From all articles, the control group's genotype frequencies were examined for deviations from Hardy-Weinberg equilibrium proportions.

**Table 1:** Summary of characteristics of included studies.

**Reference**	**Country**	**Alcoholism**			**Control**			**HWE**
		**TT**	**TC**	**CC**	**TT**	**TC**	**CC**	**p value**
Ilveskoski et al. (28)	Finland	109	12	0	44	14	0	0.883
Lappalainen et al. (23)_set1	USA	273	34	0	194	8	0	0.900
Lappalainen et al. (23)_set2	USA	144	16	0	194	8	0	0.900
Zhu et al. (24)_ Finnish	Finland	121	14	0	188	24	1	0.900
Zhu et al. (24)_Swedish	Sweden	433	39	0	156	21	0	0.883
Mottagui-Tabar et al. (29)	Sweden	531	50	0	156	21	0	0.883
Kovanen et al. (25)	Finland	459	52	1	439	67	5	0.883
Bhaskar et al. (7)_Kota	India	45	30	5	75	36	4	0.900
Bhaskar et al. (7)_Badaga	India	75	5	0	58	3	0	0.900
Akel et al. (30)	Turkey	75	5	2	75	5	1	0.248
Sengul et al. (26)	Turkey	118	5	0	150	9	0	0.900

### Statistical Analysis

The Cochran's Q test and Higgins and Thompson inconsistency I-squared statistics were used to determine heterogeneity. The association between rs16139 polymorphism and alcoholism was assessed by determining the odds ratios (OR) and 95% confidence intervals (CI) limits. As the homozygous mutant allele is rare and not present in all studies, only the allelic and dominant effects were analyzed. Overall Pooled effects and 95% confidence intervals were estimated and presented as a forest plot. To know each study's influence on the overall effect size, sensitivity analysis was conducted using a “leave-one-out” meta-analysis. It estimated the ORs for the remaining studies. A Begg's funnel plot and an Egger's regression test were used to assessing potential publication bias. For conducting the meta-analysis and constructing all plots, MetaGenyo web tool software was used (31).

## Results

### Characteristics of Included Studies

The search criteria and study selection process is depicted in Figure 1. Searching databases identified a total of 172 articles. After excluding 82 duplicate studies, 88 articles were used for further evaluation. After reading the titles and abstracts, 51 irrelevant articles were excluded, and 37 full papers were chosen for further review. Finally, eight case-control studies that met inclusion criteria were selected for data extraction (7, 23–26, 28–30). Three papers included 2 sets of data each (7, 23, 24). The genotype distributions in both alcoholism and control groups are summarized in Table 1. In total, 5,306 cases and 3,912 controls were included in the present meta-analysis. Significant between-study heterogeneity was found in both allelic and dominant genetic models (allele model: P_heterogeneity =_ 0.003, I^2^ = 63%; dominant model: P_Heterogeneity_ = 0.003, I^2^ = 62.5%). Hence, a random-effects model was used for the pooled analysis.

### Pooled Analyses for *NPY* RS16139 Polymorphism

**Figure 2:**
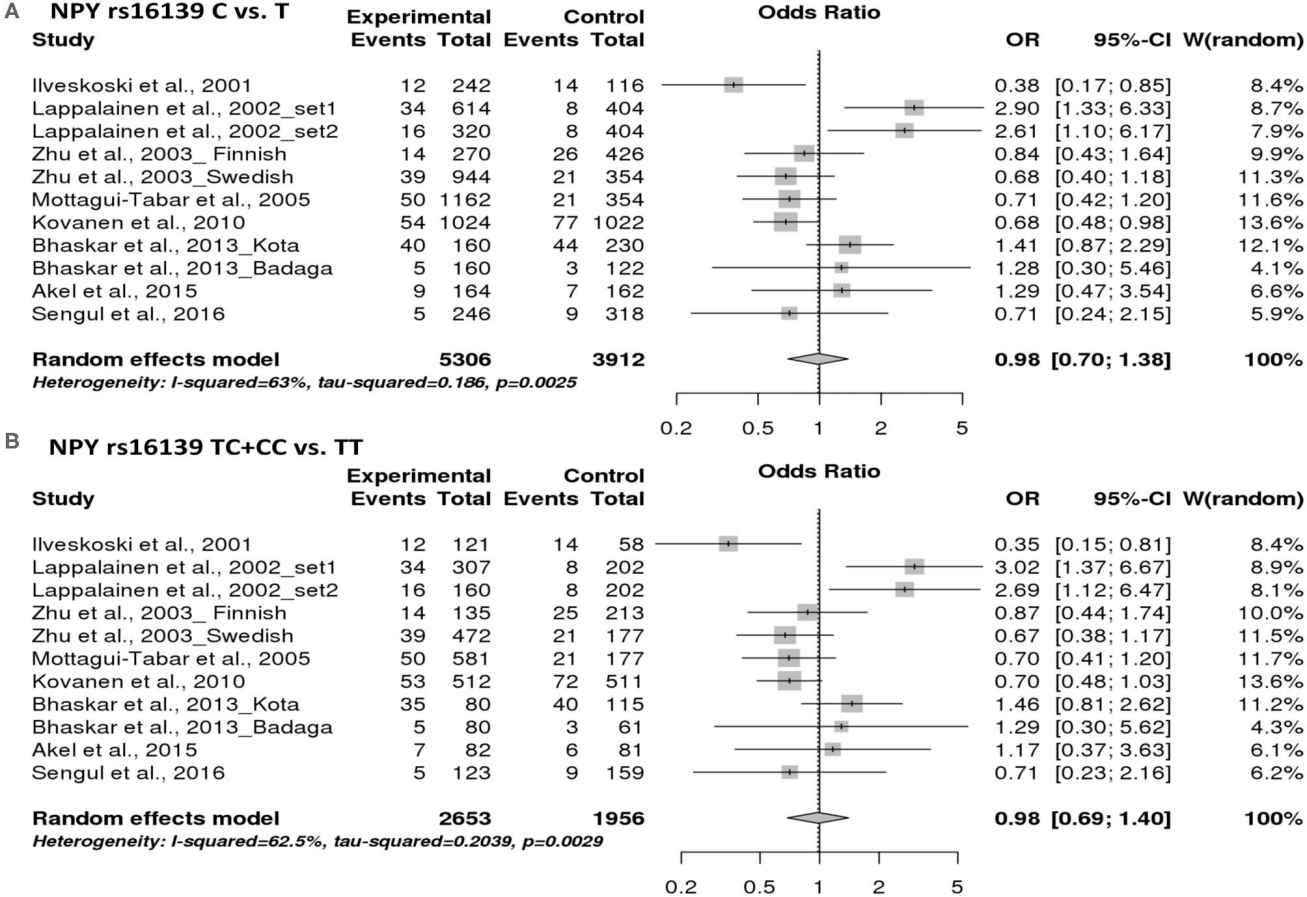
Forest plot of the association between NPY rs16139 variant and risk of alcoholism in allelic model **(A)** and dominant models **(B)**.

In order to find the association between *NPY* rs16139 variant and the risk of alcoholism, 11 studies were included in the pooled analysis. Our results have shown that there is no significant association between the risk of alcoholism and *NPY* rs16139 variant in allelic (OR = 0.98, 95% CI 0.70–1.38, *p* = 0.921) and dominant models (OR = 0.98, 95% CI 0.69–1.40, *p* = 0.919) (Figure 2).

### Sensitivity Analysis and Publication Bias

**Figure 3:**
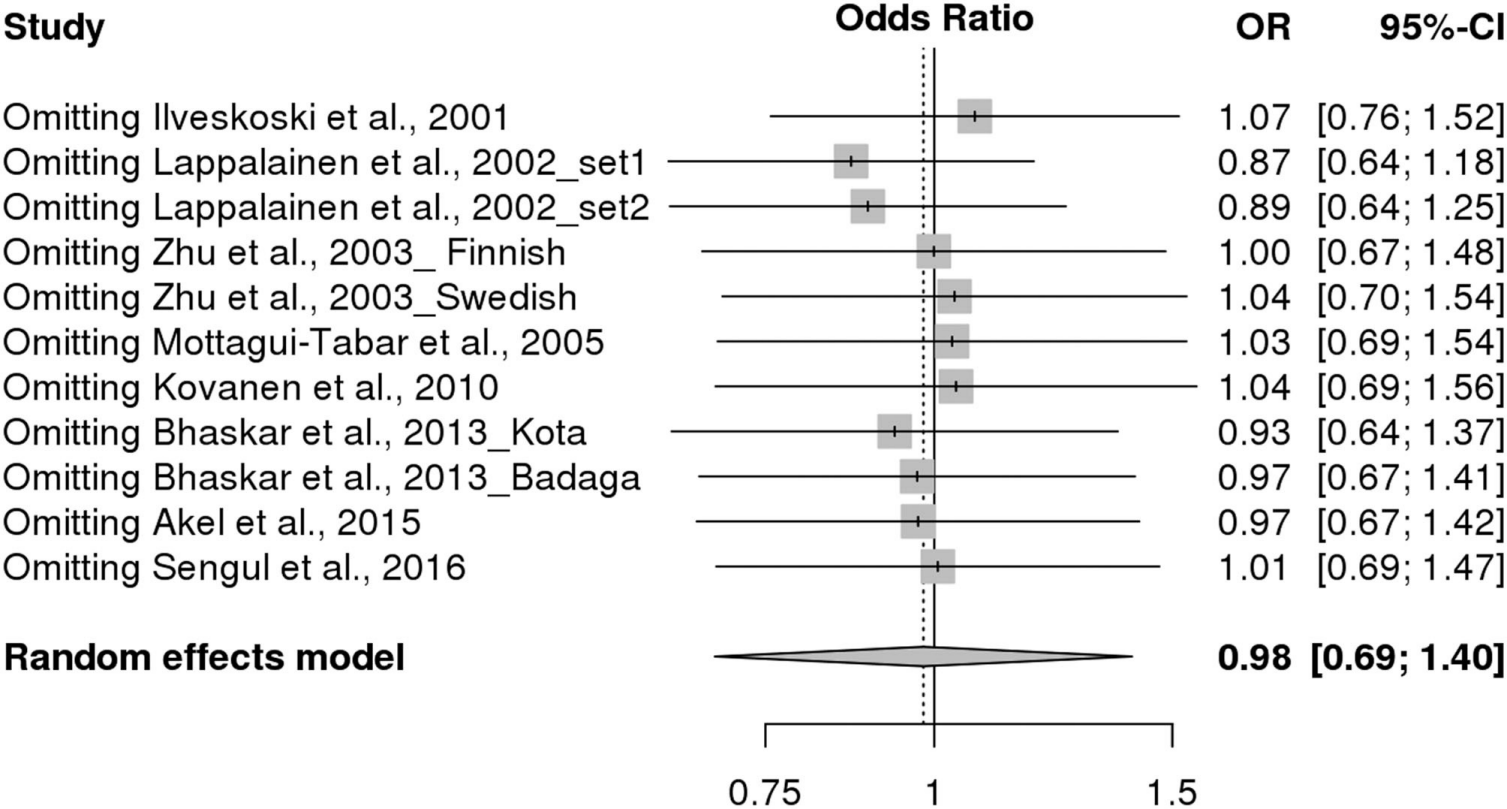
Sensitivity analysis of this meta-analysis.

**Figure 4:**
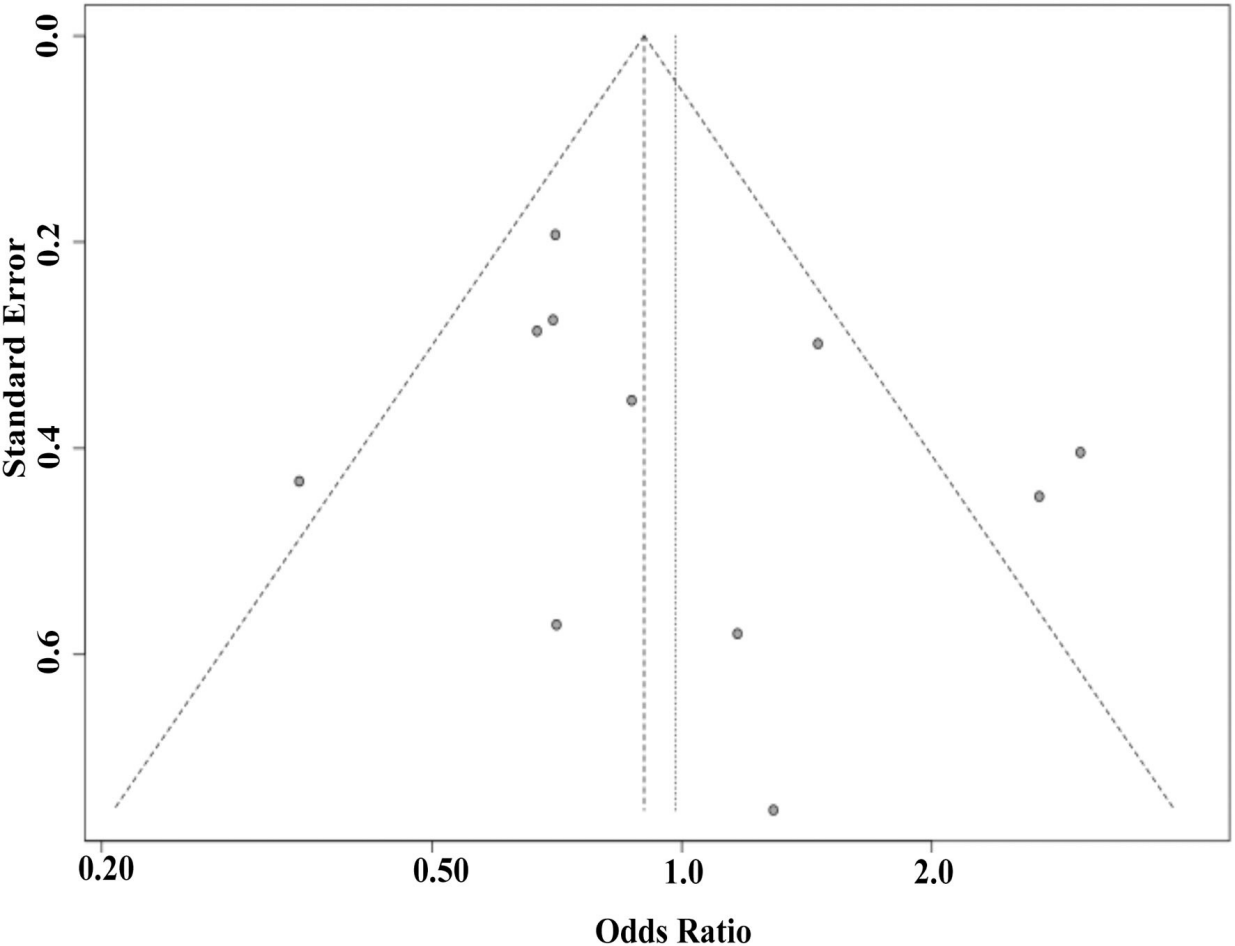
Begg's funnel plot estimating the publication bias.

Sensitivity analysis was conducted to identify the effect of each study on pooled estimates. Sensitivity analysis did not reveal any qualitative changes in pooled ORs, indicating that the results of this meta-analysis are robust (Figure 3). For *NPY* rs16139 variant, the shape of Begg's funnel plot did not reveal any evidence of publication bias (Figure 4). In addition, Egger's test revealed that there was no publication bias (*p* = 0.332).

## Discussion

The present study found no significant relation between NPY rs16139 variant and alcoholism in either allelic or dominant models. Although there is significant heterogeneity across studies, sensitivity analysis showed that the results of this meta-analysis are robust. Furthermore, there is no evidence of publication bias.

The relationship between NPY and alcohol consumption has primarily been studied in animals. Based on animal studies, the Pro7 allele has been linked to increased alcohol consumption in humans. A large body of research suggests that the NPY system, including NPY receptors, is involved in developing alcohol and drug use disorder, stress management, and anxiolysis (11). The “Pro7” allele of the rs16139 polymorphism in the NPY gene has been linked to increased mature NPY processing and higher NPY levels in cerebrospinal fluid (32). The “Leu7” allele has been shown to be rare in a depression population and to play a protective role against depression (33, 34). The first evidence for the role of the *NPY* Pro7 variant in regulating human alcohol consumption has been found in Eastern Finland, where individuals with the Pro7 variant showed >34% mean alcohol intake compared to the controls (35). Subsequent studies in Americans and European people also stated that the *NPY* Pro7 allele is more common in alcohol-dependent individuals (23). Further, the low frequency of the pro7 allele had a protective role against alcohol dependence in Finnish patients diagnosed with alcohol use disorder (28). In contrast, some other studies reported no association between *NPY* pro7 allele and alcohol dependence (24) or alcohol withdrawal symptoms (36). Analysis of three promoter polymorphisms and rs16139 could not detect positive correlations with alcohol dependence in the German population (37). The *NPY* Pro7 allele has been extremely rare and exhibits restricted distribution (38, 39). However, one of our previous studies shows the presence of Pro7 allele in many Indian populations (40).

Although the current meta-analysis's findings do not match physiological predictions, decreased expression of NPY has been observed in the alcohol-dependent individuals and during Alcohol withdrawal syndrome (AWS) (41, 42). Later, a systematic analysis of the NPY gene and its receptor revealed that polymorphisms in the NPY gene are not linked to alcoholism or AWS (43). There is significant heterogeneity across studies included in this meta-analysis. In some studies, the controls were social drinkers; in some others, controls were derived from the general population. There is also likely to be heterogeneity in the diagnosis of phenotypes across studies. The *NPY* Pro7 allele shows the discontinuous distribution in mixed ethnicities due to geographically variable selection; the present meta-analysis results have limited applicability.

## Conclusion

Despite considering some limitations, Accumulating evidence of the NPY system may offer an attractive target for developing novel therapies for alcohol dependence. However, the present meta-analysis suggests that NPY rs16139 polymorphism is not associated with alcoholism. To the best of our knowledge, this is the first meta-analysis to examine the link between the NPY rs16139 and the risk of alcoholism. For the precise results, further studies with large-scale animal and human models are needed to increase our understanding of the relationship between NPY variants in alcoholism.

## Data Availability Statement

The original contributions presented in the study are included in the article/supplementary material, further inquiries can be directed to the corresponding authors.

## Author Contributions

BC, MY, WY, and LB contributed to conception and design of manuscript. MY and MM searched, screened the articles and extracted data. MY and LS performed the data-analyses. BC, HV, and LB provided additional suggestions and assisted in the interpretation of data. BC and MY drafted the manuscript. WY and LB critically revised the manuscript. All authors contributed to the article and approved the submitted version.

## Conflict of Interest

The authors declare that the research was conducted in the absence of any commercial or financial relationships that could be construed as a potential conflict of interest.

## Publisher's Note

All claims expressed in this article are solely those of the authors and do not necessarily represent those of their affiliated organizations, or those of the publisher, the editors and the reviewers. Any product that may be evaluated in this article, or claim that may be made by its manufacturer, is not guaranteed or endorsed by the publisher.
